# Paradoxical response during antituberculous therapy in a patient discontinuing infliximab: a case report

**DOI:** 10.1186/1752-1947-3-6673

**Published:** 2009-04-01

**Authors:** Young Kyung Yoon, Jeong Yeon Kim, Jang Wook Sohn, Min Ja Kim, Ja Seol Koo, Jai Hyun Choi, Dae Won Park

**Affiliations:** 1 Division of Infectious Diseases, Department of Internal Medicine, College of Medicine and Institute of Emerging Infectious Diseases, Korea University, South Korea; 2 Department of Internal Medicine, Institute of Digestive diseases and Nutrition, Korea University Medical Center, South Korea; 3 Division of Infectious Diseases, Department of Internal Medicine, Ansan Hospital, Korea University, South Korea

## Abstract

**Introduction:**

The use of the drug infliximab for the treatment of patients with Crohn's disease can be complicated by tuberculosis. A paradoxical reaction during antituberculosis chemotherapy and immunologic reconstitution after discontinuation of infliximab can result in severe disseminated tuberculosis.

**Case presentation:**

A 38-year-old Korean man with severe Crohn's disease presented with fever and diffuse abdominal pain. Infliximab had been started 2 months before admission. A chest X-ray and abdominal computed tomography scan revealed numerous miliary nodules in both lung fields and microabscesses in the spleen. Given the diagnosis of disseminated tuberculosis, the infliximab therapy was discontinued and antituberculosis therapy was promptly started. Over the next 3 months, the patient was diagnosed with tuberculosis lymphadenitis on a right supraclavicular lymph node and surgical excision of the lesion was performed. With the diagnosis of a paradoxical response, anti-tuberculous therapy was continued for 12 months.

**Conclusion:**

Our case suggests that patients who develop tuberculosis after infliximab exposure are at an increased risk of developing a paradoxical reaction. The current recommendation of discontinuing infliximab during tuberculosis treatment should be re-evaluated.

## Introduction

In 1998, the drug infliximab, developed from a murine-human chimeric anti-tumor necrosis factor (TNF) monoclonal antibody that binds diverse TNF moieties, was approved by the US Food and Drug Administration for the treatment of Crohn's disease [1]. However, infliximab poses a risk for reactivation of latent granulomatous infections by disrupting established granulomas; this occurs due to the neutralization of soluble TNF that is essential for the formation and maintenance of a granuloma [2]. As a result, granulomas fail to serve as physical barriers to mycobacterial dissemination in patients with latent tuberculous infections.

A paradoxical deterioration during anti-tubercular therapy is defined as a transient worsening of disease, at a pre-existing site, or the development of new tuberculous lesions in a patient who initially improved on anti-tubercular therapy. This phenomenon is more commonly associated with extrapulmonary tuberculosis [3]. A paradoxical exacerbation of the signs and symptoms of tuberculosis may occur not only after tubercular therapy, but also after discontinuation of TNF-α inhibitors during the treatment of active tuberculosis, due to an enhanced antituberculous immune response.

Although there is ample data suggesting an association between treatment with infliximab and the development of tuberculosis, there has been some debate on the treatment of paradoxical reactions and disseminated tuberculosis in patients treated with infliximab [4]-[6]. Here, we describe a patient who became systemically ill with disseminated *Mycobacterium tuberculosis* infection, and had a paradoxical response to discontinuation of TNF-α inhibitors during anti-tuberculous therapy.

## Case presentation

**Figure 1:**
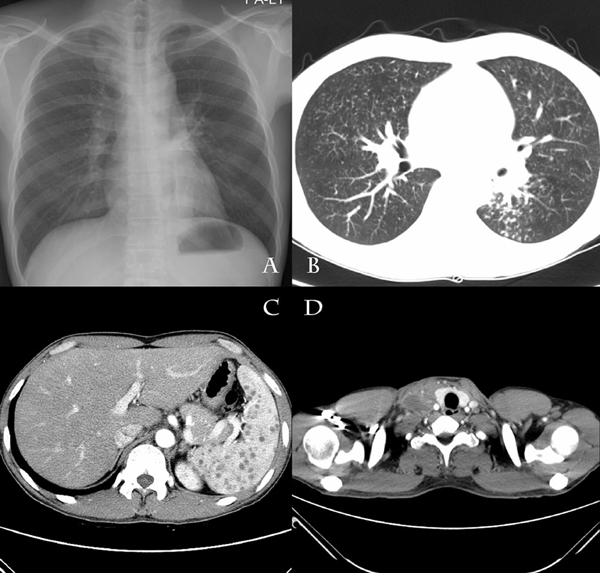
**Chest X-ray and computerized tomography scans of the abdomen and neck**.

The patient was a 38-year-old Korean man with severe Crohn's disease that had been diagnosed by clinical features and colonoscopy findings 9 years previously. The patient had been hospitalized with a 2 week history of fever and diffuse abdominal pain. He had been started on infliximab (Remicade®, Centocor) which had been given at baseline and at 2 and 6 weeks. Infliximab infusion therapy was performed three times because of an aggravated Crohn's disease activity index and unresponsiveness to high-dose steroids (methylprednisolone 60mg). The tuberculin skin test was negative, and chest X-rays were normal before the infliximab administration. Three weeks later, the patient started to complain of fever, diffuse abdominal discomfort and fatigue. On admission, his temperature was 39 degrees Celsius, pulse rate 100/min, respiratory rate 24/min and blood pressure 100/80mmHg. Routine physical examination did not show any physical abnormalities except for diffuse abdominal tenderness without rebound tenderness. Laboratory investigations revealed anemia (Hct 31.1%, Hb 9.89g/dL), an elevated C-reactive protein level (9.1mg/L), an elevated erythrocyte sedimentation rate (90mm/h) and no other abnormal findings. Chest X-ray and abdominal computed tomography (CT) scan (Figure 1a), performed shortly after admission, revealed numerous miliary nodules in both lung fields and multiple tiny low density nodular lesions in the spleen that were consistent with microabscesses (Figure 1b and 1c). The tuberculin skin test on admission was positive (16mm). Induced sputum was obtained and acid-fast bacilli (AFB) staining and cultures for *Mycobacterium tuberculosis* were positive.

Histologic examination of a lung-tissue sample obtained by percutaneous needle biopsy showed a chronic caseating granulomatous inflammation. In addition, AFB staining of lung tissue revealed acid-fast bacilli. Polymerase chain reaction confirmed an infection with *M. tuberculosis*. The tuberculosis culture confirmed *M. tuberculosis* which was susceptible to all anti-tuberculous drugs. Infliximab therapy was discontinued, and antituberculosis therapy (isoniazid, rifampin, pyrazinamide and ethambutol) was promptly started after confirmation of the diagnosis. After 2 days of therapy, the fever resolved and then the other symptoms progressively improved.

Over the next 3 months, the patient was compliant with the antituberculous medication. However, he presented with a painful swelling of a right supraclavicular lymph node with redness and warmth at the site of the lesion. We performed a CT scan of the cervical region and thorax that showed poorly enhanced lesions in the right upper mediastinum, upper and lower paratracheal, retrosternal region and left supraclavicular fossa consistent with tuberculosis lymphadenitis (Figure 1d). Surgical excision of the supraclavicular lymph node was performed due to complaints of pain and purulent discharge from a fistula. Histologic examination of the lymph node samples revealed chronic caseating granulomatous inflammation, but the AFB staining and culture were negative. The diagnosis of a paradoxical response was made; isoniazid, rifampin and ethambutol were continued without change of the treatment regimen. The patient completed a 12-month course because miliary nodules and splenic microabscess were still noted on a follow-up CT scan. His Crohn's disease was controlled with mesalazine, after remission had been achieved with the three cycles of inflixmab.

## Discussion

Paradoxical worsening during therapy for tuberculosis is a well-recognized phenomenon. A paradoxical reaction, or immune reconstitution, occurs more commonly in patients infected with human immunodeficiency virus (HIV), who are simultaneously treated with antiretroviral therapy [7]. In this case, the newly developed supraclavicular tuberculous lymphadenitis may have represented a similar immune reconstitution after recovery from iatrogenic immunosuppression. The patient was treated with surgical resection of the lesion without modification of the antituberculous regimen because of complaints of pain and fistula formation.

Paradoxical response does not warrant more drugs or longer medication. Many studies have shown that utilising rifampin, isoniazid, pyrazinamide and ethambutol for the initial phase, followed by rifampin and isoniazid for a further continuation phase, should be the recommended standard treatment for adult pulmonary or extra-pulmonary tuberculosis. It is now well established that ethambutol can be omitted in patients with a low risk of resistance to isoniazid according to the recommendations by the World Health Organization [8]. But the Korean Academy of Tuberculosis and Respiratory Disease (1997) and the Korean Centers of Disease Control and Prevention (2005) recommend the ethambutol combination for patients with drug susceptible tuberculosis, because the resistance rate to isoniazid is more than 4% in Korea. The new guidelines of the Korean Academy of Tuberculosis and Respiratory Disease (2005) argue that a regimen of rifampin and isoniazid in continuation phases is sufficient for patients with drug-susceptible tuberculosis. Hence the need for ethambutol in the continuation phase of chemotherapy can still be optional in cases in Korea.

Our patient received three doses of infliximab after negative findings of both tuberculin skin test screening and chest X-ray screening. However, before the screening tuberculin skin test, the patient received a high dose of steroid treatment, which might have affected the result of the tuberculin skin test. A false negative tuberculin skin test has been previously reported during immunosuppression with prednisone, azathioprine and infliximab [6]. TNF-α, an inflammatory cytokine expressed by activated macrophages, T-cells and other immune cells, plays a crucial role in host responses against tuberculosis, including granuloma formation and inhibition of dissemination [9]. Thus, patients with tuberculosis who lack adequate TNF-α production are at risk for disseminated, rapidly progressing and unusual presentations of tuberculosis. Infliximab is a chimeric antibody against TNF-α, resulting in the loss of the ability by macrophages to sequester mycobacteria through phagocytosis, as well as failure of induction of mycobacterial eradication [9]. Although the clinical efficacy of infliximab is well established, there are many case reports and studies showing an increased risk of tuberculosis in patients treated with infliximab [4,10].

Paradoxical reactions generally occur within 1 to 3 months of initiation of treatment. Several theories of pathogenesis have been proposed for paradoxical tuberculous reactions, yet the precise mechanisms remain to be defined. One theory to explain paradoxical responses is an excessive inflammatory response in the context of immune reconstitution and increased antigen exposure after tuberculosis therapy [11]. Why paradoxical reactions to drug therapy should occur only in some individuals is unclear; however, it is likely to be due to a complex interplay of the host immune responses, tubercle bacilli virulence, antigen load, the site of infection and the effects of the chemotherapy. Paradoxical reactions with tuberculosis have been well studied in patients with HIV infections. The massive delivery of membrane antigens, after the initiation of antituberculosis treatment, has been advocated as the cause of paradoxical responses in immunocompetent patients [12]. However, in HIV-infected patients, immunologic restoration after antiretroviral therapy, and recovery of specific responses to certain antigens, is another possible mechanism [11].

In our case, an increased antigen exposure after tuberculosis chemotherapy, and immunologic reconstitution secondary to the discontinuation of infliximab, might be implicated in the development of a paradoxical reaction, similar to patients infected with HIV. The current recommendation is for the discontinuation of TNF-α inhibitors in patients during treatment for active tuberculosis; this is mandatory for those patients with proven mycobacterial infections [12,13]. However, there is no contra-indication to systemic glucocorticoid therapy in patients with tuberculosis being treated with infliximab [13]. Glucocorticoid therapy is recommended in patients with tuberculous meningitis or pericarditis. The outcomes with other immunosuppressive agents during the treatment of tuberculosis have been variable. Studies on tuberculosis in organ transplant recipients suggest that that chances of survival are not decreased by the use of cyclosporine or azathioprine [14]. In addition, reports on the treatment of tuberculosis associated with HIV have shown that highly immunosuppressed patients respond well to standard tuberculosis therapy; some clinicians recommend withholding the initiation of antiretroviral therapy until the completion of the intensive phase of tuberculosis treatment [15]. Other reports suggest, however, the maintenance of low doses of a TNF-α inhibitor or to continue the TNF-α inhibitor therapy during treatment of active tuberculosis; at present, the safety implications of either approach are unclear [4]-[6,13]. However, this approach to treatment should be considered because the immunologic regulation could provide control of Crohn's disease and be beneficial in these patients.

## Conclusion

Our case suggests that patients who develop tuberculosis after infliximab exposure are at increased risk of a paradoxical reaction. Therefore, the current recommendation for the discontinuation of TNF-α inhibitors in patients with Crohn's disease during the treatment of active tuberculosis should be re-evaluated. Additional studies are required to evaluate the safety and efficacy of continuing TNF-α inhibitors during tuberculosis therapy.

## Abbreviations

AFB: acid-fast bacilli; CT: computed tomography; HIV: human immunodeficiency virus; TNF: tumor necrosis factor.

## Consent

Written informed consent was obtained from the patient for publication of this case report and accompanying images. A copy of the written consent is available for review by the Editor-in-Chief of this journal.

## Competing interests

The authors declare that they have no competing interests.

## Authors' contributions

JHC and JSK were involved in diagnosis of the case and preparation of the manuscript. JYK, JWS and MJK advised on the management of the patient and assisted in editing the manuscript. YKY and DWP drafted the manuscript and were involved in patient management and follow-up. All authors read and approved the final manuscript.
